# Gender differences in the subjective evaluation of factors determining human health in Russians

**DOI:** 10.1192/j.eurpsy.2022.1396

**Published:** 2022-09-01

**Authors:** R. Shilko, L. Shaigerova, O. Almazova, A. Dolgikh, M. Rabeson

**Affiliations:** Lomonosov Moscow State University, Psychology, Moscow, Russian Federation

**Keywords:** gender, factors determining human health, subjective evaluation, sociocultural determination

## Abstract

**Introduction:**

Research into sociocultural mediation of human health engages the role of gender differences in the subjective evaluation of factors that determine health status.

**Objectives:**

The focus of the research was the respondents’ opinion about the importance of various factors for human health and subjective well-being.

**Methods:**

210 men and 403 women aged 14 to 76 years (M = 26.9; SD = 13.7) from six regions of the Russian Federation participated in the study. Participants were asked to rank six factors: genetics, healthy lifestyle, good ecology, regular medical examination, absence of stress (ability to cope with them), financial well-being in terms of their impact on human health (1 is the most important, 6 is the least important).

**Results:**

Both men and women consider “healthy lifestyle” to be the most important factor for human health, while financial well-being - most unimportant. Using the t-test for two independent samples, it was found that: women consider “absence of stress (the ability to cope with it)” significantly more important for health than men (t = -2.569; p = 0.010), while men consider “financial well-being” to be significantly more important than women (t = 2.807; p = 0.005).

**Conclusions:**

It was revealed that men and women equally indicate the most and least important factors determining health. At the same time, subjective assessments of the importance of such factors as absence of stress and financial well-being for health have significant differences. The reported study was funded by the RFBR, project number 17-29-02506.

**Disclosure:**

No significant relationships.

